# Tuberculosis preventive therapy in Sierra Leone: early implementation gaps and lessons learned

**DOI:** 10.5588/pha.26.0005

**Published:** 2026-05-18

**Authors:** L. Farma-Grant, J.S. Kanu, B. Molleh, I.F. Kamara, A.R.Y. Kamara, N. Sesay, W.K. Lahai, B. Fofanah, M.-A. Falama, O.K. Offe Amponsah, M. Mahmoud, D.S. Grant, Y. Siatty, R. Zachariah, J. Koroma, M. Mustapha, T. Sesay, S. Kenneh

**Affiliations:** 1 Ministry of Health, Freetown, Sierra Leone;; 2 National Public Health Agency, Freetown, Sierra Leone;; 3 College of Medicine and Allied Health Sciences, University of Sierra Leone, Freetown, Sierra Leone;; 4 UNFPA Country Office, Freetown, Sierra Leone;; 5 World Health Organization, Country Office, Freetown, Sierra Leone;; 6 Department of Pharmacy Practice, Faculty of Pharmacy and Pharmaceutical Sciences, Kwame Nkrumah University of Science and Technology, Kumasi, Ghana;; 7 The National Leprosy and TB Programme, Ministry of Health, Freetown, Sierra Leone;; 8 UNICEF, UNDP, World Bank, WHO Special Programme for Research and Training in Tropical Diseases (TDR), Geneva, Switzerland;; 9 KYM Consultancy, Freetown, Sierra Leone.

**Keywords:** tuberculosis, TPT, contact tracing, community health workers

## Abstract

**BACKGROUND:**

In 2023, Sierra Leone started tuberculosis preventive therapy (TPT) implementation. The tuberculosis programme recommends four contacts per index case for tracing and screening.

**METHODS:**

This study was to assess the performance of early implementation of contact tracing, screening, and TPT uptake in five regional hospitals.

**RESULTS:**

Of 905 index cases recorded, 1,222 contacts were traced and screened by community health workers. Of these, 433 were enrolled into TPT, and 353 (82%) completed the TPT regimen. Eighty out of 353 (18%) on TPT were lost to follow-up.

**CONCLUSION:**

Deficiencies in documenting contacts and logistical constraints to trace, screen, and follow up for enrolment and completion of TPT need to be addressed for TPT scale-up.

Tuberculosis (TB) remains a disease of significant global concern; with an estimated 10.6 million infections globally, and 1.6 million deaths in 2022.^1^ Sub-Saharan Africa continues to be a hotspot for TB, having 60% (18/30) of countries with high TB burden. There is good evidence to indicate high TB transmission among household contacts of TB cases.^2,3^ To reduce transmission and infection, the WHO recommends countries treat latent TB for priority groups like people living with HIV, and provide TB preventive therapy (TPT) for household contacts of TB patients, including children below 5 years.^1,4^ Early identification, contact screening, and implementation of TPT have proven effective in reducing TB infection by preventing progression from latent TB to active disease, decreasing transmission, and mortality.^1,4,5^

Sierra Leone is a high-TB-burden country.^1^ The National Leprosy and TB Programme started implementing TPT in 2023 for priority groups and household contacts,^1,6^ setting a target of 90% by 2025. Despite gains made by the programme in 2022 in the identification of index cases and a reduction in mortality, the programme still faces challenges in the early diagnosis of index cases, contact tracing and screening, and the implementation of TPT.^6,7^ When index cases are confirmed at the health facility, contacts of those index cases are to be line listed by the health care worker. Tracing and screening of contacts are done by community health workers (CHWs) in the community. Those presumptive for TB during screening (TB symptom screening guidelines) are sent for confirmation at the health facility and those non-presumptive are sent for enrolment and initiation of TPT at the health facility. Only 14% of health facilities are offering TB services nationwide.^6–8^ The programme has observed teething problems with the TPT implementation, and they decided to map them out and address them in their next strategic plan (2026–2030).^8^ Scaling up TPT in the country without identifying and addressing these underlying hurdles could compromise TB treatment, accelerate resistance development, and pose public health risks. The situation may be similar in other countries embarking on the same and these insights might have wider ramifications.

We therefore conducted an assessment of the TPT implementation in five referral hospitals.

## METHODS

This was a descriptive study of early cascade performance and implementation bottlenecks based on patient records review. Sierra Leone is a small country in West Africa and is divided into five administrative regions: North, South, East, North-West, and Western Area regions. We reviewed TPT registers at the five main referral hospitals in the five regions: regional hospitals in Makeni (North), Bo (South), Kenema (East), Port Loko (North-West), and Connaught Teaching Hospital Complex (Freetown Western Area Urban).

We included all index cases for TB from July to December 2024. Data collectors used the EPIcollect5 for recording data. Summary data were collected on index cases, their contacts who were traced and screened, those enrolled into TPT, the type of regimen, and status of treatment. Linking contacts (those traced and screened) and those with presumptive TB (from symptoms) to their index cases was difficult; this was due to the use of multiple registers, the cadre of staff entering the data, and the stage at which data are entered into the TPT implementation care system.

Data were summarised using frequencies, proportion, and ratios. Furthermore, the TPT cascade was presented in programmatic themes, implementations steps, challenges at each stage, and the possible recommendations (Table 1).

**TABLE 1.** TPT implementation flow, challenges, and recommended actions in five regional districts of Sierra Leone (July–December 2024).

Programmatic theme	Implementation steps	Key challenges	Recommended actions	Responsibility level for actions
1. Investigation of the index case and contact documentation	• Index case properly investigated, and number of contacts recorded at the health facility.	• No record of contacts at the facility at the time of case confirmation.	• Ensure robust documentation of all contacts at the point of index case confirmation.	• Health facility
	• The TB programmes recommend at least four contacts to be traced and screened per index case.	• Number of contacts not communicated to CHWs for follow-up.	• Establish a standardised system to share contact lists with CHWs promptly.	• National
				• District
2. Identification of contacts of index cases in the community	• Facility HCWs provide index case information to CHWs.	• Suboptimal identification of contacts.	• Train CHWs on accurate contact line-listing.	• National
	• CHWs line-list all contacts of index cases at the community level.	• Incomplete or missing line-listing.	• Provide transport reimbursements or logistics (bicycles/motorbikes) and communication support (phones or bundles) to enhance tracing and follow-up.	• District
		• Lack of transport or communication support for CHWs.		• National
3. Screening and referral of contacts from community to the health facility	• CHWs conduct screening using cardinal TB symptoms (fever, cough >2 weeks, weight loss, night sweats).	• Inadequate training of CHWs on TB and TPT	• Include TB and TPT modules in CHW training and mentorship.	• National
	• Presumptive cases (≥2 symptoms) are referred to the health facility for confirmation.	• Lack of motivation for contacts to visit facilities.	• Facility HCWs to mentor CHWs on TB contact tracing.	• Health facility
	• Asymptomatic contacts are referred for TPT enrolment.	• No follow-up system to confirm referral completion.	• Provide incentives and or transport reimbursements to encourage contacts to visit facilities.	• National
	• Contacts traced and screened are asked to come to the facility voluntarily.	• Delays in tracing, screening, and TPT initiation.	• Strengthen referral tracking systems to ensure linkages between community and facility.	• District
		• Weak linkage between presumptive cases and index cases.		• District
		• Low number of index-to-contacts ratio seen (1:1, instead of 1:4)		
4. Enrolment and follow-up of eligible contacts on TPT (at the health facility)	• At the facility, contacts are screened, weighed, and tested for HIV before TPT enrolment.	• Loss to follow-up among contacts on TPT.	• Implement community-level TPT delivery using CHWs to enhance adherence.	• District
	• Eligible contacts are enrolled and placed on one of four regimens:	• Poor adherence and treatment completion.	• Strengthen follow-up and supervision by facility staff.	• National
	- 6H (isoniazid daily for 6 months)	• Low number of enrolment of contacts into TPT (433/1,222) contacts	• Introduce digital tracking systems or SMS reminders for missed monthly visits.	• National
	- 3RH (rifampicin + isoniazid daily for 3 months)			
	- 3HP (rifapentine + isoniazid weekly for 3 months)			
	- 1HP (rifapentine + isoniazid daily for 1 month)			
	• Monthly refills required.			
5. Documentation of TPT completion	• Contacts completing TPT are issued treatment completion cards and recorded in the TPT register.	• Poor documentation of TPT outcomes and incomplete data entry.	• Train facility nurses on proper documentation of TPT outcomes.	• National
	• Outcomes include: Completed, Loss to Follow-up, Died, or Transferred Out.	• Inconsistency in variables across the registers.	• Standardise TPT and other TB registers and ensure regular data audits.	• National
	• Currently there are multiple registers for documentation, namely: Index case, Contacts tracing (used by CHWs), the TPT register, HIV register, and Lab registers.			

Index Case (also called the source case) = the initial person in the family, group, or community who has been diagnosed with TB; Contact of an Index Case = any person who has been exposed to an individual with pulmonary TB disease like those living in the same household; Screened for TB = the process of evaluating ‘contacts TB signs and symptoms’ (cough for more than 14 days, weight loss, fever, fatigue, neck swelling, and night sweats); Presumptive Case (formerly ‘TB suspect’) = anyone presenting with signs and symptoms of TB from screening with further test required to confirm or rule out TB; Enrolled into TPT = a contact that does not have presumptive TB and started on TPT.

TB = tuberculosis; TPT = TB preventive therapy; HCW = health care worker; CHW = community health worker.

### Ethical statement

Ethics approval was obtained from the Sierra Leone Ethics and Scientific Review Committee (SLESRC Number: 022/02/2025).

## RESULTS

There were 905 index cases confirmed, and a total of 1,222 contacts were traced, identified, and screened, a ratio of 1 index case to 1 contact. Although an observed 82% completion rate (353 of 433) for contacts on TPT treatment is promising, the 18% loss to follow-up remains a major gap – especially because Kenema regional hospital, the eastern region, has the highest loss to follow-up among contacts on the TPT; despite having recorded the highest number of TB index cases (Table 2). Similarly, in high-burden settings like Pakistan and Nigeria, only 35% and 57%, respectively, of eligible contacts of index patients received timely medical evaluation and TPT initiation, due to barriers with contact identification, timely screening and TPT initiation, and health care worker knowledge.^9,10^

**TABLE 2.** Summary of index cases, their contacts, and TPT intervention in five regional referral hospitals in Sierra Leone.

Districts	Index cases	Number of contacts of the index	Ratio of index to contact	Contacts on TPT	Regimen type			LFU, n (%)^A^	TPT completed, n (%)^B^
					6H	3HP	3RH		
Bombali	185	67	3:1	67	0	67	0	6 (9)	61 (91)
Port Loko	42	110	1:3	105	80	15	10	3 (3)	102 (97)
Kenema	285	595	1:2	164	32	94	38	59 (10)	105 (64)
Bo	147	287	1:2	38	4	28	0	7 (2)	31 (82)
Connaught	246	163	10:7	59	16	38	5	5 (3)	54 (92)
**Total**	**905**	**1,222**	1:1	**433**	**132**	**242**	**53**	**80 (18)**	**353 (82)**

TPT = tuberculosis preventive therapy; 6H = isoniazid 6 months; 3HP = isoniazid 3 months; 3RH = rifampicin and isoniazid 3 months; LFU = loss to follow-up.

A LFU: Row percentage with contact on TPT as denominator.

B TPT completed: Row percentage with contact on TPT as denominator.

## DISCUSSION

This is the first study to assess TPT implementation in Sierra Leone. These findings are relevant, more so when the TB programme established a targeted uptake of 90% and an expected index-to-contact case ratio of 1:4.^7^ The study found a low index-to-contact ratio of 1:1 and a high loss to follow-up after TPT initiation, weakening the preventive impact of TPT in reducing TB incidence and mortality especially in high-risk groups, creating an apparent risk for the emergence of drug-resistant TB. Importantly, the findings point to deep-rooted health system challenges affecting TPT delivery, including limited knowledge among community and facility-based health workers, supply inconsistencies, multiple and burdensome recording tools, delays in screening and initiation, and patient-level barriers such as inadequate information and difficulty accessing health facilities.^8^ These findings are likely to reflect the operational reality of implementing TPT across various levels of health care facilities in Sierra Leone, shaped by geographic distribution of facilities, mixture of services, and the traditional referral systems.

## CONCLUSION

Harmonisation or digitalisation of TPT data tools, strengthening of CHWs and health care workers knowledge, and improving logistics for contact tracing and follow-up are essential for scale-up.
